# Building management capability for clinical veterinary organisations—An Australian pilot study

**DOI:** 10.1002/vro2.70007

**Published:** 2025-02-12

**Authors:** Zhanming Liang, Taleta Hompas

**Affiliations:** ^1^ College of Business, Law and Governance Townsville Queensland Australia

**Keywords:** competency assessment, leadership, management competency, veterinary

## Abstract

**Background:**

Veterinary care is facing critical levels of attrition that challenge its sustainability in the provision of standards of care. A competent and skilled management workforce, along with enhanced organisational structures and procedures, is essential to effectively address the challenges that veterinary organisations face today and in the future.

**Methods:**

The pilot study adapted the Management Competency Assessment Partnership (MCAP) tool to collect data from 35 mid‐level and senior managers working in five veterinary organisations in Australia via an online survey to understand their competency development needs and the obstacles that they were facing in the management roles. Univariate analyses, Pearson correlations, Kaiser‒Meyer‒Olkin test and Bartlett's test of sphericity were performed.

**Results:**

This study confirmed that the MCAP tool maintained good internal consistency and identified competency gaps that managers in the five veterinary organisations should consider addressing. The study confirmed the positive correlation between informal management‐related training and self‐study on management issues and self‐perceived management competency level. The results supported the need for veterinary organisations to provide management training to foster a culture of continuous improvement and life‐long learning among veterinary managers.

**Conclusions:**

This study highlighted the core elements essential for the building management capacity of veterinary care services and organisations. It also validated the value of management competency self‐assessment in identifying the development needs of managers, demonstrating how the management development framework adapted from the human healthcare sector can guide the development of a competent management workforce for veterinary care.

## INTRODUCTION

Veterinary care is facing unprecedented challenges, such as governmental underinvestment, low remuneration, unsustainable level of burnout, stress and negative mental health outcomes among veterinary professionals.^1^, ^2^, ^3^, ^4^, ^5^ With the high attrition and increased demand for animal care and challenges facing veterinary services, improved organisation efficiency as well as skilled and capable managers who can effectively develop and implement strategic solutions and manage the provision of quality animal care are critical. Evidence from the human medical profession has suggested the important roles that competent managers play in organisation success, quality care provision^6^, ^7^ and improved staff retention.^8^, ^9^ The evidence also reinforces that developing and improving managers’ capability and enhancing organisation's management capacity are worthwhile.^10^, ^11^, ^12^ It is reasonable to expect that investments in developing and equipping managers with the capacity to find sustainable solutions to the new and emerging strategic challenges facing veterinary care, both now and into the future, are necessary.

**FIGURE 1:**
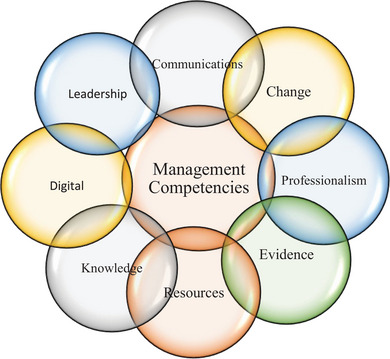
Core management competencies for health service managers.

Previous studies have reported on some of the management skills that are essential for veterinary managers, including critical thinking, communication and interpersonal skills, decision making, emotional intelligence, leading change, leadership, team building skills and strategic planning.^2^, ^3^, ^13^, ^14^ The comprehensive skills and knowledge requirements necessary for veterinary managers to successfully fulfil their managerial roles have not been developed. Management competency frameworks, similar to those developed in the human medicine context that may guide the development of the management curriculum, have yet to be developed in the veterinary care context. Some examples of such frameworks developed in the human healthcare context are the Healthcare Leadership Model by the MHS Leadership Academy (UK), the Australasian College of Health Service Managers Competency Framework (Australia), the Health Leadership Competency Model (USA) and the International Hospital Federation Leadership Model (Switzerland).^15^, ^16^, ^17^, ^18^, ^19^ Management competencies commonly identified as core for human health service managers are shown in Figure 1, which are adapted from a scoping review,^20^ and the seventh competency of ‘Digital—demonstrated understanding of digital health and digital management’, which is part of the validated Management Competency Assessment Partnership (MCAP) tool.^21^, ^22^, ^23^

Clear competency requirements and guidance in developing the capability of leaders and managers working in veterinary care are required. It is particularly important when management positions in veterinary organisations are often filled by clinical staff based on clinical performance rather than management competence.^5^, ^24^ Veterinary professionals who are clinically competent may lack the non‐clinical skills, knowledge, aptitudes and attitudes that are correlated with economic success.^24^ Management skills are required at every level of the veterinary profession to enable veterinary services to respond to changing societal expectations and evolving new technologies, provide high‐quality animal care and retain their position as leaders in the community and society. A clear leadership and management transition is required to prepare veterinary professionals to take on management roles.^1^, ^5^ Improving veterinary organisations' support to their managers is equally important as veterinary managers often take on management roles with adequate training and preparation, finding the management roles callenging.

An online survey was conducted and there were three questions.

1. Can competency development needs of managers working in veterinary organisations be identified via a self‐management competency reflection process?
2. What are the difficulties commonly encountered by managers working in veterinary organisations and the preferred support from veterinary organisations?
3. Is there a positive correlation between training and self‐perceived competency?

## METHODS

A cross‐sectional online survey was conducted in Australia to answer the above research questions between November 2023 and March 2024. Senior and mid‐level managers working at the following five veterinary organisations were invited to participate in the online survey.

1. James Cook University (JCU) Vet, emergency small animal care (https://jcuvet.com.au/) affiliated with JCU.
2. Tropical Vets, all species animal care (https://tropicalvets.com.au/).
3. Animal Emergency Services, emergency small animal care at seven locations (https://animalemergencyservice.com.au/).
4. Veterinary Specialist Services, specialised in small animal care (https://vss.net.au/).
5. Small Animals Specialist Hospitals, specialised in small animal care (https://sashvets.com/).

These five veterinary organisations were selected for their representation of emergency, specialised care, small animal care and emergency hospital care. Apart from JCU Vet and Tropical Vet, which are local to northern Queensland, the other three are national organisations with operations in different Australian states.

## SAMPLING AND RECRUITMENT

There were approximately 105 senior and mid‐level managers working in the five veterinary hospitals/clinics who were all invited to participate in the study. The director of each organisation emailed the invitation together with the participant information sheet and a link to the online survey to their senior and mid‐level managers. Implied consent was included on the front page of the survey. The purpose and significance of the study, as well as its potential impact on veterinary care and veterinary management, were outlined in the email. The information would help managers to make an informed decision about their voluntary participation. No financial compensation or other incentives were offered. Given the small number of managers in each of the five veterinary organisations, participants were not asked to disclose the type of organisation that they worked for ensuring the voluntary nature of the study.

With a 95% confidence interval and 5% margin of error, also assuming that 85% of managers would score 4 or above on competency (as informed by previous MCAP studies), a minimum of 35 veterinary managers were required. This sample size was calculated using https://abs.gov.au/websitedbs/D3310114.nsf/home/Sample+Size+Calculator. According to the Central Limit Theorem, a sample size of over 30 was considered sufficiently large to address the survey questions. Furthermore, considering that the minimum sample size for a Spearman correlation test to obtain reliable results was 30, 35 or more valid online survey completions were regarded as adequate for the study.

## ONLINE SURVEY QUESTIONNAIRE

The questionnaire for the online survey was adapted from the MCAP management competency survey originally developed and validated in the Australian human healthcare system^16^, ^21^ and adapted and validated in other countries such as China, India and Iran.^20^, ^25^, ^26^, ^27^ Each of the competency behavioural items was carefully reviewed and revised to match the veterinary context when appropriate. It was then reviewed by a veterinarian who had completed a Master of Business Administration qualification to ensure its content validity. Each questionnaire took approximately 25 min to complete and consisted of the following quantitative components (Supporting Information S1).

1. Demography, educational background and previous and current work experience.
2. Management‐related training undertaken.
3. Difficulties encountered in the management roles (17 difficulties were provided for ranking using a five‐point Likert's frequency scale—never, rarely, sometimes, often and all the time).
4. Psychological empowerment^28^ (12 items were assessed using a five‐point Likert's agreement scale).
5. The importance of six core management competencies (from the MCAP tool) using a five‐point Likert's importance scale.
6. Acquisition of the six core competencies prior to taking up the current management role using five‐point Likert's agreement scale.
7. Self‐perceived competency level of six core competencies and the associating 82 behavioural items (from the MCAP tool) using the seven‐point descriptive competency scale, as detailed in Table 1.^21^

**TABLE 1:** Management Competency Assessment Partnership tool—competency descriptive scale for self‐assessment.^21^

Scale^a^	Level	Competency level
1	Not competent	Do not understand the requirements and am not capable of applying it to my role
2	Basic or novice	May be capable of demonstrating minor aspects in my role
3	Advanced beginner	May be capable of demonstrating in my role, but not in all required aspects
4	Competent with occasional guidance	Can generally demonstrate in my role, but guidance is needed occasionally
5	Competent, no guidance	Can demonstrate in my role independently without guidance, but have not had extensive experience
6	Proficient	Always apply appropriately in my role with extensive experience
7	Superior expertise	Always apply appropriately in my role with extensive experience gained from diverse management roles at executive level and can teach this competency to others

^a^ Scores less than 5 were considered less than fully competent. Scores of 5 or greater were considered fully competent.

The survey questionnaire also included the following three open‐ended questions:

1. Their career goals and how the organisation can best support them in achieving the goals.
2. Obstacles in demonstrating the core competencies in the management roles.
3. Preferred support in developing and demonstrating management competency in management positions.

In the first page of the online survey, an explanation of the purpose of the study, instructions of survey completion and consent to participate, with assurance of identity protection, were included.

## CORE MANAGEMENT COMPETENCIES INCLUDED IN THE MCAP TOOL

C1 Evidence: Evidence‐informed decision making (13 behavioural items).

C2 Resources: Operations, administration and resource management (17 behavioural items).

C3 Knowledge: Demonstrated knowledge of environment and the organisation in the veterinary industry (11 behavioural items).

C4 Communications: Interpersonal, communication qualities and relationship management (19 behavioural items).

C5 Leadership: Leading people and organisations (13 behavioural items).

C6 Change: Enabling and managing change (nine behavioural items).

The Qualtrics survey platform (https://qualtrics.com/) was used to host the online questionnaire.^21^, ^29^

### Data management and analysis

The online survey data were downloaded in MS Excel format (Microsoft) from the Qualtrics website and checked for completion and error. Questionnaires with missing data from the first five components of the survey were excluded from analysis. All the data were analysed using IBM SPSS Statistics (version 29.0). The mean of the six competencies and the combined mean for each of the six competencies based on the associating behavioural items were calculated. When examining the competency development needs of a management group, it usually looks at how many have received a competency score 3 or less (indicates incompetent), 4 (competent but requiring occasional guidance) and 5 or more (competent). The percentage of managers in each of these competency score group were also calculated.

Frequencies of selection for the ‘difficulties’ and ‘management training’ and mean score of each of the four dimensions for psychological empowerment were calculated. Univariate analyses, including tests for normality, were carried out. Correlations between self‐perceived management competency level and management/non‐management training, self‐study on management‐related topics and years as a manager were tested by Pearson correlation with alpha level of significance set at 0.05.

Correlations between self‐perceived management competency level and management/non‐management training, self‐study on management‐related topics and years as a manager were tested via Pearson correlation to determine the effect size, the *r* coefficient, which measures strength of the relationship between the tested variables. Kaiser‒Mery‒OIkin (KMO), Bartlett's test of sphericity and Cronbach's alpha coefficient were performed to confirm MCAP tool's internal consistency and MCAP data's suitability for factor analysis. Content analysis was conducted on the qualitative data collected from the questionnaire after it was downloaded into Excel in order to identify emerging themes.

## RESULTS

A total of 35 questionnaires were included in the data analysis presenting a response rate of around 34%. An additional 12 managers attempted the survey but did not complete all questions in the first five components. Hence, their entries were excluded from the analysis.

### Demography, employment, education and training

Ten out of the 35 managers (28.6%) possessed either a master's degree (*n* = 6) or a doctorate degree (*n* = 4). Seven versus 18 managers possessed a technical and further education qualification or bachelor's degree as their highest qualification, respectively. The number of years as a manager ranged between 2 and 22 years (mean = 6.37; medium = 4); years in the current management position ranged between 1 and 6 years (mean = 2.64; medium = 2). Five out of the six managers who possessed a master's degree had been managers for 7 years or more. All of the managers who possessed a PhD had been a manager for less than 3 years. Only two of the degrees were management related (Master of Business Administration). When asking participants whether they committed 10 h per year in the past 3 years in various trainings and self‐studies, 18 managers (51%) said yes to both non‐management and management training, and another six managers (17%) attended either non‐management or management training. Eighteen managers (51%) said yes to self‐study on management‐related topics, among which 15 (43%) also said yes to either non‐management or management training. Three managers indicated that they did not participate in any form of training or self‐study.

### Difficulties encountered in the management positions

The difficulties that managers had encountered in their management roles were selected as either ‘often’ or ‘all the time’ by more than one‐third of managers as follows:

1. Time constraint (92.7%)
2. Balancing dual clinical and management responsibilities (65.9%)
3. Team conflict (48.8%)
4. Confronting higher management level/dealing with conflicting priorities of senior management (39.0%)
5. Peer conflict (36.6%)
6. Having to learn something new such as information or medical technology (36.6%)
7. Employee engagement in decision making and implementation of change (34.1%)

### Psychological empowerment

The 12 items can be grouped into four themes: meaning (items 1‒3), competence (4‒6 items), self‐determination (7‒9 items) and importance (10‒12 items). The mean scores and standard deviation (st) for the four themes were 4.5 (st = 0.612), 3.9 (st = 0.769), 3.7 (st = 0.852) and 3.8 (st = 0.858), respectively.

### Perceived importance, acquisition and self‐assessment of management competencies

Between 80% and 88% of managers rated each of the six competencies as being important or very important. A much lower proportion of the managers agreed or strongly agreed that they had acquired the competencies prior to taking up the management positions: C1 Evidence (51%), C2 Resources (34%), C3 Knowledge (63%), C4 Communications (71%), C5 Leadership (63%) and C6 Change (37%).

### Self‐assessment of overall competency level (using the seven‐point descriptive competency scale)

**FIGURE 2:**
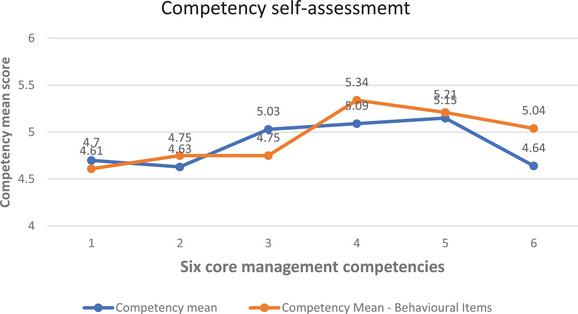
Competency mean scores comparison.

**FIGURE 3:**
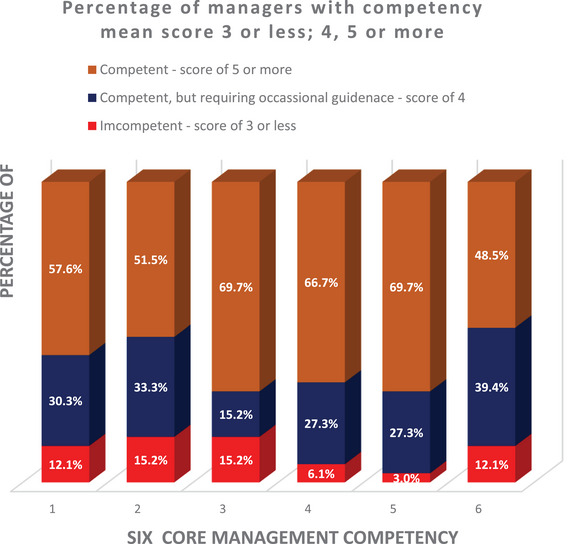
Proportion of managers versus self‐assess competency score groups.

The mean scores for each of the competencies generated directly from assessing the competencies themselves or the associating behavioural items are shown in Figure 2. Figure 3 shows the proportion of participants who gave themselves scores for each of the six competencies in three competency score groups.

### Self‐assessment of 82 behaviour items (using the seven‐point descriptive competency scale)

**FIGURE 4:**
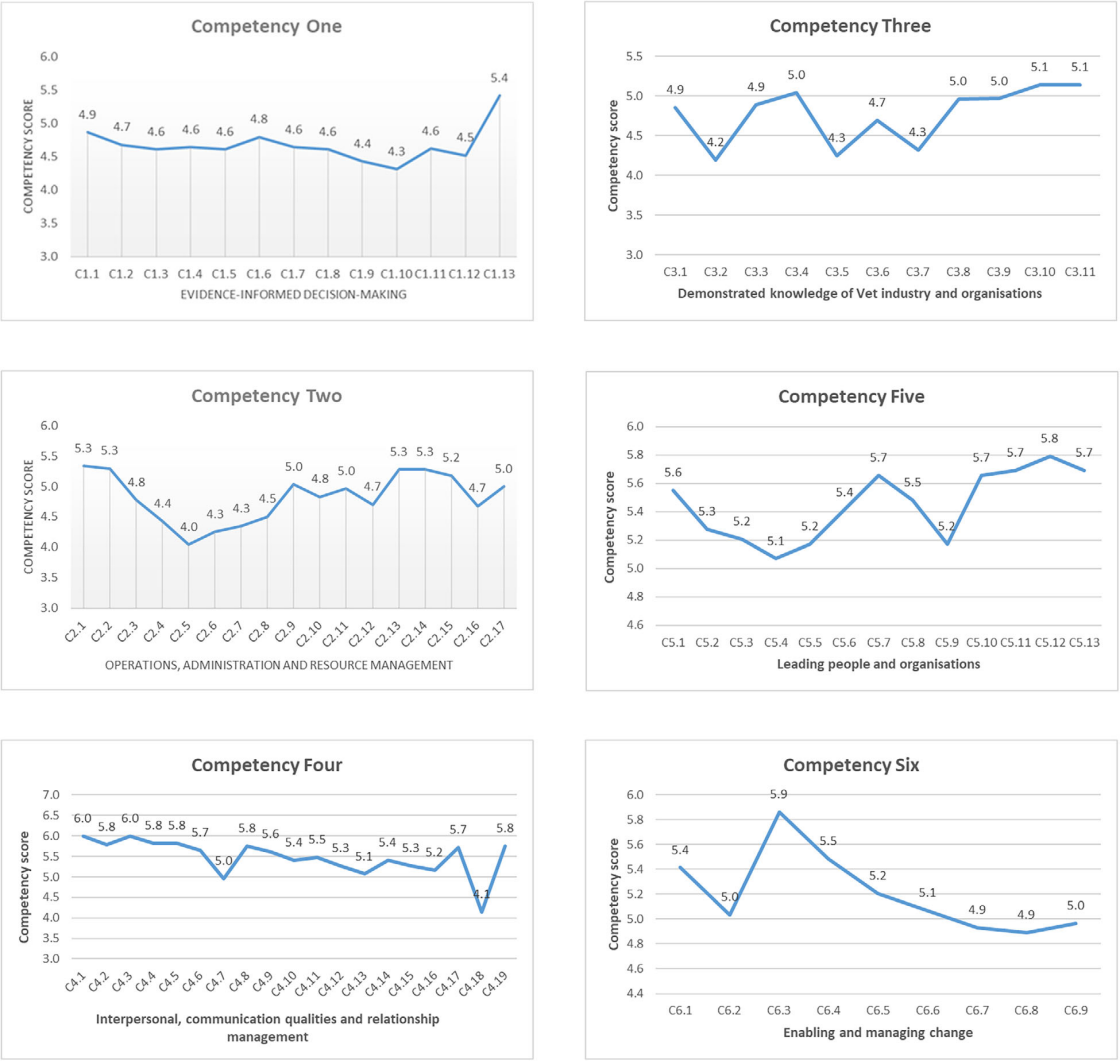
Mean scores of behavioural items for each of the six competencies.

Figure 4 shows the mean score for each of the behavioural items associating with each of the six competencies. There is variation in the mean score received for each behavioural item. Higher or lower scores (being 0.5 or more lower than the highest score of the relevant competency) indicated strengths versus weaknesses for the competency respectively.

Reviewing the behavioural items that received scores lower than the associating competency mean, the areas requiring development and improvements were as follows.

- Application of relevant legislation and quality indices to guide quality service provision and improvement
- Developing and managing budget
- Developing contingency plan
- Engaging key stakeholders in service design and decision making
- Financial management
- Implementing change and evaluating change outcomes/benefits
- Investing time for self‐care, mental health and developing supportive networks
- Managing and solving conflicts
- Performance management—measuring performance and conducting performance review
- Setting and using measures to evaluate decision outcomes
- Understanding the external context/environment and its impacts on veterinary organisations

### Internal consistency and suitability for factor analysis

The KMO measure of sampling adequacy for each of the six competencies yielded values greater than 0.7, indicating that the competency data were suitable for factor analysis. Additionally, the Cronbach's alpha coefficient for each competency exceeded 0.7, demonstrating that the MCAP scale exhibited good internal consistency. All scores are detailed in Table 2.

**TABLE 2:** Results on internal consistency and suitability for factor analysis testing.

Competency	One	Two	Three	Four	Five	Six
KMO value	0.835	0.758	0.829	0.836	0.857	0.766
Bartlett's test of sphericity						
Approximate chi‐square	470.466	476.062	254.528	540.744	355.293	176.004
df	78	136	55	171	78	36
*p*‐Value	<0.001	<0.001	<0.001	<0.001	<0.001	<0.001
Cronbach's alpha	0.952	0.912	0.911	0.944	0.937	0.890

Abbreviation: KMO, Kaiser‒Mery‒OIkin.

### Correlation between competencies, training and years as a manager

**FIGURE 5:**
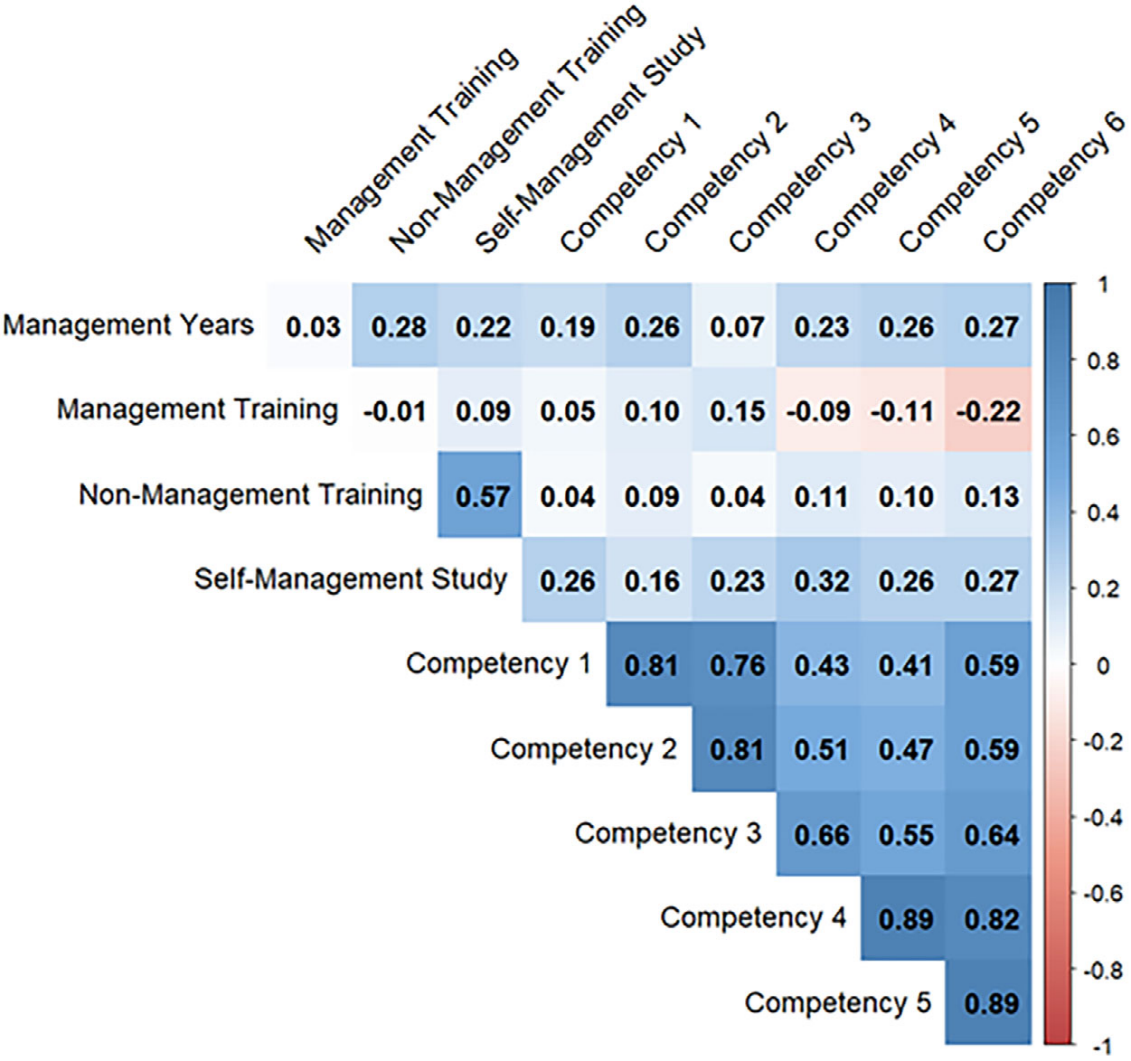
Heatmap of Pearson correlations (*r*) between comparison groups.

Correlations between competencies, management training, non‐management training, self‐management study and years as managers were tested and are shown in Figure 5. Figure 5 is a heatmap with the colours of blue and orange relating to positive or negative associations between two variables, respectively, with the values of the correlation output. In addition, the ‘darkness’ of that colour is related to increased correlation numbers. The heatmap (Figure 5) illustrates the strong correlations between competency groups.

Nearly all competencies had an *r*‐value greater than 0.5, suggesting a strong positive relationship. There was a positive correlation between management years, non‐management training and self‐study on management topics versus all competencies.

### Correlation between competency and psychological empowerment

Correlations between each of the management competencies and the four different dimensions of psychological empowerment, namely, meaning, competence, self‐determination and importance, were also tested. The results are included in Table 3.

**TABLE 3:** Correlations between competency and four dimensions of psychological empowerment.

Competency mean score	Meaning	Competence	Self‐determination	Importance
C1	−0.200	0.543^**^	0.265	0.412^*^
C2	−0.146	0.511^**^	0.207	0.31
C3	−0.011	0.457^*^	0.312	0.418^*^
C4	0.119	0.662^**^	0.477^**^	0.288
C5	0.127	0.722^**^	0.467^*^	0.366
C6	−0.039	0.649^**^	0.493^**^	0.358
Six competencies combined	−0.037	0.675^**^	0.411^*^	0.409^*^

* *p* < 0.05.

** *p* < 0.01.

‘Competence’ was positively correlated with all core competencies, with competencies 4, 5 and 6 exhibiting higher correlation coefficients (*r*‐values) compared to competencies 1, 2 and 3. Additionally, ‘competence’, ‘self‐determination’ and ‘importance’ all had positive correlations with the combined competencies. The *r*‐values for ‘meaning’ were mostly negative, but these correlations were small in magnitude, suggesting that they may not truly represent a negative relationship and could be influenced by the small sample size.

### Organisational support in demonstrating management competency and achieving career goals

Thirty‐one managers shared their views on how organisations could best support them in achieving their career goals and developing and demonstrating competency in their management roles. The following three categories emerged during content analysis.

1. Decision making and autonomy
  - Better consultation and clearer strategic direction from head office, with more inclusion in decision‐making processes.
  - Fewer bureaucratic hurdles (‘less red tape’) to move forward with initiatives that had already been approved.
  - Enabling independence and autonomy in decision making.
2. Leadership and support
  - Providing coaching, on‐the‐job experience and training, and regular feedback, particularly radical feedback focused on practical improvements.
  - Regular support and backing from senior leaders.
  - Clearer expectations and processes within the organisation to streamline operations and decision making.
  - Providing role clarity and performance expectations.
3. Enhancing capability
  - Better recognition of skills and experience to facilitate career progression.
  - Providing leadership and management training.
  - Providing more opportunities for managers to engage in broader roles, beyond just scheduling and team management.
  - Regular access to network‐wide training, workshops and online courses.

Allowing sufficient time to balance management and clinical tasks was also mentioned by several managers.

## DISCUSSION

The study not only confirmed the relevance of the management competencies identified in the human healthcare sector to veterinary managers, but also supported the relevance of core management competencies regardless of sectors and management levels, as previously reported.^17^, ^30^, ^31^ The study also confirmed that the internal consistency of the MCAP tool remained when used by managers from non‐health sector, indicating the suitability of adapting the MCAP tool in the veterinary care context in identifying competency gaps and development needs of veterinary managers. The MCAP tool identified both the strengths and weaknesses of managers’ competencies. It highlighted those with urgent management competency development needs (scoring lower than 4) and those requiring occasional assistance (scoring between 4 and lower than 5), ensuring that they can continue to deliver satisfactory management performance.^22^, ^26^, ^27^ It is alarming that between 30% and 51% of managers gave themselves a competency score lower than 5 across the six core management competencies. A competency score of less than 5 indicates that managers are not able to demonstrate their competency independently and are not well equipped to fulfill their management roles.^16^, ^21^, ^29^ This confirms that a large proportion of the veterinary managers participating in the study require assistance to comprehensively develop their managerial competencies.

The results from the current study and studies of mid‐level human healthcare managers, using the MCAP tool,^25^, ^26^, ^27^, ^32^, ^33^ suggest that the need for upskilling and supporting veterinary managers is greater than managers working in the human health sector. Experience from other industries demonstrates that targeted management training and professional development opportunities can enhance management competency,^34^, ^35^, ^36^ improve managers’ engagement with staff,^37^ foster teamwork and collaborative partnerships, enhance patient care and service quality,^38^ and boost productivity and efficiency.^39^ Studies in human healthcare confirm a positive correlation between management competency of healthcare leaders, their education attainment, and participation in leadership and management‐related training and seminars,^40^, ^41^, ^42^ which is consistent with the findings from the current study. The key competency development areas outlined in the ‘Results’ section could provide an evidence‐based foundation for designing and delivering training to meet the competency development needs of veterinary care managers.

Although the pilot study was not designed to generalise its findings, it confirmed a positive correlation between self‐perceived management competency level and participation in informal management‐related training and self‐study of management issues. This confirms the applicability of evidence from studies in human healthcare,^35^, ^36^, ^37^ which also provides evidence for the needs within veterinary organisations and professional institutions to offer management training and foster a culture of continuous improvement and life‐long learning among veterinary managers in the five selected veterinary organisations. Further study is underway, aiming to involve a larger number of managers from various veterinary organisations to generate evidence that supports more generalisable conclusions for guiding the system‐level development of the veterinary management workforce. Additional study is needed to ascertain the value of embedding management competency assessment into the annual management appraisal process, providing objective evidence to inform the formulation of professional growth plans and demonstrating organisational commitment to staff development and support.

Both the qualitative and quantitative data collected from the study identified common difficulties encountered by participating veterinary managers, similar to those reported in the healthcare settings using the MCAP tool. These challenges include time constraint, new skill acquisition and conflicts with peer, team members and higher management level.^25^ The current study highlighted the difficulties specific to veterinary management roles, such as balancing dual clinical and management responsibilities.^5^, ^24^ As noted earlier, in the veterinary care organisations, mid‐level managers are often appointed to the management role without management training or sufficient management experiences, making it difficult to navigate dual management and clinical responsibilities. This issue is not unique to veterinary care because similar concerns have been raised in studies of hospitals providing human healthcare.^43^, ^44^, ^45^ Moreover, the lack of training and professional development opportunities, along with limited access to on‐the‐job training, coaching and mentoring were also identified as key challenges in the current study.

**FIGURE 6:**
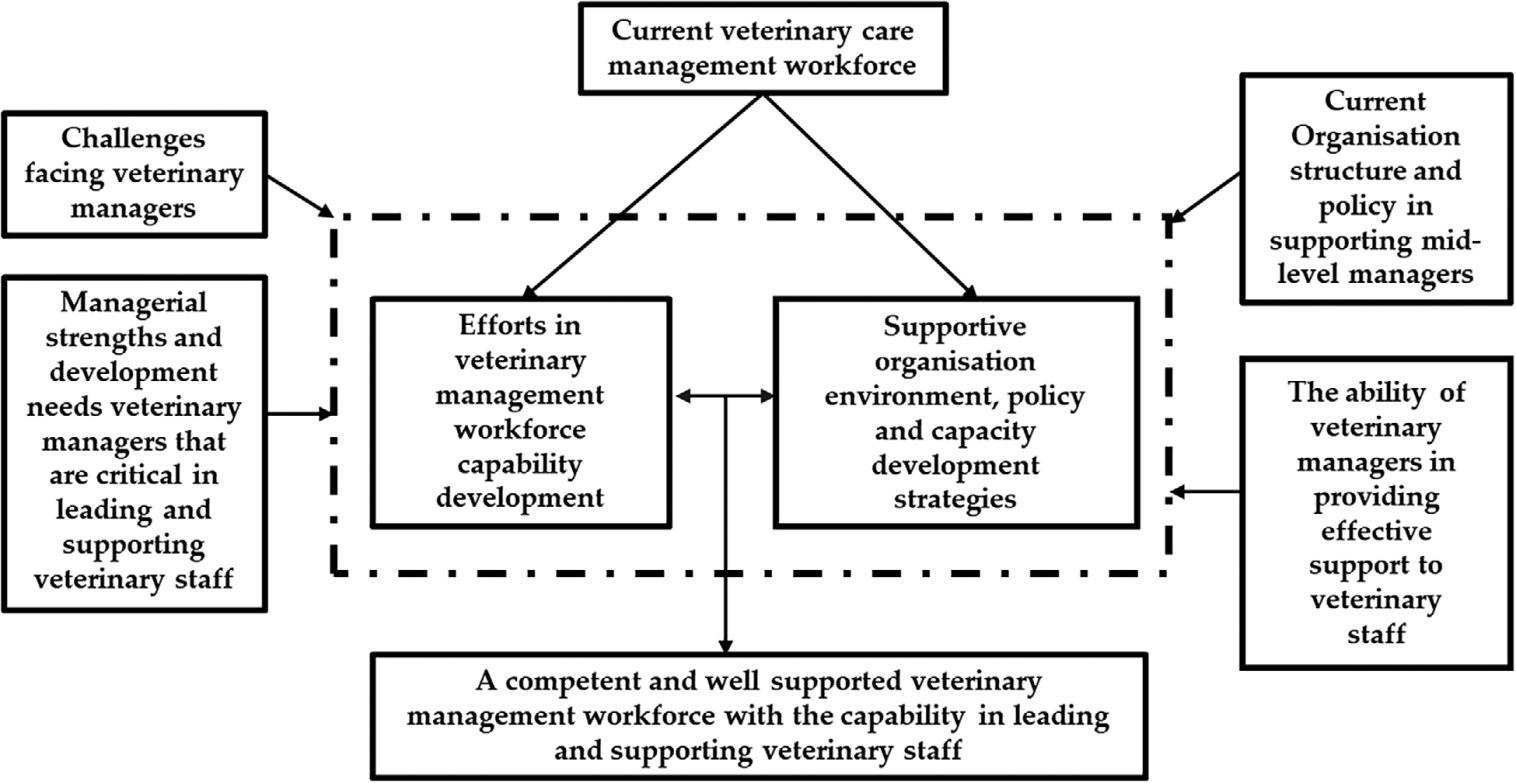
Framework for developing management workforce for veterinary care (as adapted from Ref. 23).

An organisational capacity building model emphasised that staff capability development, role clarity, organisation structures and processes, and supervision and support mechanism are core competencies for enhancing organisation capacity in terms of service efficiency and effectiveness.^46^ The preferred organisational support identified in this study aligns with the capacity building model.^46^ Investment in continued professional development, leadership and management training, and adequate organisational support, such as mentoring, coaching, effective feedback and role clarity, is necessary to enhance managers’ capabilities in fulfilling their managerial roles, thereby contributing to staff retention and job satisfaction.^10^, ^11^, ^12^ Evidence from the human healthcare sector supports the notion that improved management capability boosts managers’ confidence in leading and managing teams and organisations successfully.^26^ Such investment demonstrates an organisation's commitment to supporting staff's skill development, career advancement and empowerment, which ultimately improves staff job satisfaction and retention.^12^, ^47^ Based on the learnings from previous studies and findings from current study, Figure 6 proposes a management development framework for veterinary care, adapted from the healthcare industry.^23^

The pilot study confirmed the relevance of the core management competencies identified in the healthcare sector to veterinary care, as well as the applicability of the MCAP tool in identifying competency development needs of veterinary managers. The study also provided evidence to guide the revision of the list of challenges most pertinent to veterinary management, which will be incorporated into revisions of the MCAP survey instrument. This study has helped construct a list of appropriate support mechanisms that veterinary care organisations can provide to enhance veterinary managers’ ability to develop and demonstrate their management competencies.

The main limitation of this study was related to sampling and sample size. While 35 participants are sufficient for a pilot study, the sample size is too small to account for the influence of factors such as geographic locations, organisation type, investment in management development, management levels and types of management positions on self‐perceived management competencies. A larger‐scale study is needed to draw generalisable conclusions that can inform system‐level changes aimed at enhancing management capabilities in veterinary care. Although managers were encouraged to participate in the study by the organisations, lack of compensation of the time and efforts that managers needed to put into the completion of survey may potentially lead to self‐selection bias among managers. For example, managers who were familiar with and/or had interests in developing management competencies or professional development might be more likely to volunteer themselves for the study. Additionally, some terminology commonly used in healthcare, such as ‘quality indices’ and ‘stakeholders’, may not be familiar to veterinary managers. Including further definitions in the MCAP survey for veterinary managers should be considered. Although research evidence has proven the value of self‐assessment/self‐reflection in identifying individual's development needs, including supervisor, peer and direct report in the assessment will add objective evidence to the assessment process, which can be considered in future studies.

The pilot study confirmed the need to enhance veterinary managers’ capability and highlighted the core elements essential for building management capacity within veterinary care services and organisations. It underscored the importance of veterinary organisations and professional institutions in providing management training and fostering a culture of continuous improvement and life‐long learning among veterinary managers. The study validated the value of management competency self‐assessment in identifying development needs of managers and demonstrated how the management development framework, adapted from the healthcare sector, can guide the creation of a competent management workforce in veterinary care. The MCAP competencies and the refined management competency assessment tool can be used to set clear competency development goals for managers working in veterinary organisations.

## AUTHOR CONTRIBUTIONS

Zhanming Liang was responsibile for the conceptualisation and design of the study and the initial drafting of the manuscript. Taleta Hompas drafted the abstract, conclusion, and part of the introduction. Both Zhanming Liang and Taleta Hompas provided critical review, revision, and editing of the manuscript and approved the final revision before submission.

## CONFLICTS OF INTEREST

The authors declare they have no conflicts of interest.

## FUNDING INFORMATION

The authors received no specific funding for this work.

## ETHICS STATEMENT

Ethics approval was granted by James Cook University Human Ethics Committee (approval ID: H9259). All participants consented to participate in the study prior to proceeding to complete the survey.

## Supporting information



Supporting Information

## Data Availability

The datasets used and/or analysed during the current study are available from the corresponding author upon reasonable request.
