# Supplementary material for: Building management capability for clinical veterinary organisations—An Australian pilot study
Source: Vet Rec Open. 2025 Feb 12;12(1):e70007. doi: 10.1002/vro2.70007 (PMC11814534; doi:10.1002/vro2.70007)
Supplement: Supplementary file 1 — Supporting Information [file VRO2-12-e70007-s001.pdf]

JAMES COOK UNIVERSITY AUSTRALIA

Introduction

.

Welcome to Leadership and Management Capacity Building in the Veterinary Industry Survey

@Zhanming Liang

Purpose of the survey

You are invited as leaders and managers within the Pilot Project Industry Partners - Veterinary hospitals / clinics to participate in this online survey aiming to achieve the following:

1) confirm core competency requirements for managers in the veterinary industry;

2) confirm competency gaps and competency development needs of leaders and managers working in the vet hospitals / clinics;

3) identify challenges facing management competency development and demonstration, and

4) seek suggestions of how best the organisation can support their leaders and managers.

What details are included in the assessment?

- Demographic, educational background and work experience.
- Six management competencies (82 items) – using MCAP Tool (Liang et al. 2018).
- Psychological Empowerment (12 items) – using Spreitzer Psychological Empowerment Scale (Spreitzer, 1995).
- Difficulties encountered in your management roles.
- Four open-ended questions to seek your thoughts on how to develop management competencies and how best the organisation can support you as a manager with the Veterinary industry.

How long will each assessment take?

Approximately 30 minutes for Self-Assessment. The assessment is set up in a way allowing you to log on or log off multiple times and modify the answers before submission.

What do you need to do before commencing the assessment?

- fully understand the purposes of the assessment;
- fully understand the MCAP 7-point competency descriptive scale to be used to assess the competencies;

- accept that the assessment is anonymous;

Please contact A/Professor Zhanming Liang on zhanming.liang@jcu.edu.au or Dr Taleta Hompas on taleta.hompas@jcu.edu.au should you require further information or confidential discussions.

I am sure that you will find the assessment process beneficial in improving your understanding of management competency requirements.

Associate Professor Zhanming Liang and Dr Taleta Hompas
College of Public Health, Medical and Veterinary Science
James Cook University

1.

Informed Consent

Before proceeding the survey, you need to provide consent to the participation in this project.

You understand that by selecting 'yes, you give your consent to the participation of the survey', you confirm that details of the project have been clearly explained to you and you have been provided with a written information sheet to keep.
You understand that the researcher may use the results as described in the information sheet.

You acknowledge that
1) taking part in this study is voluntary and you are aware that you can stop taking part at any time without explanation or prejudice and can request the withdrawal of any unprocessed data that you have provided. Researcher will identify the survey that you completed by the code that you created and entered at the beginning of the survey. Such code is only recognised by yourself.
2) no names will be used to identify you with this study without your approval

- ○ Yes, I give my consent to the participation of the survey
- ○ No, I would not like to participate in the survey

2.

Please type in a code that can help us to 'find' your completed survey for you when necessary.

[ ]

2a. Please select the organisation that you are currently employed.

- ◯ SASH (Small Animal Specialist Hospital)
- ◯ AEA (Animal Emergency Australia)
- ◯ VSS (veterinary Specialist Services)
- ◯ Tropic Vets
- ◯ JCU Vet Hospital

---

3. Have many years have you been in the current management position?

---

4. How many years have you been working as a manager (including the current management position) in the vet industry?

---

5. Do you have any of the following degrees? Please select all that apply and also provide the discipline of each of the degree programs completed in the box provided.

- ☐ TAFE
- ☐ Bachelors
- ☐ Master
- ☐ Doctorate or PhD

---

6. Are you currently studying to obtain a degree? If yes, please provide the type of degree and the discipline area of study?

- ◯ No
- ◯ Yes

6a. I am currently studying for the following degree (please also provide the discipline of degree programs in the box provided).

- ◯ TAFE
- ◯ Bachelors

- ◯ [ ] Master
- ◯ [ ] Doctorate or PhD

Second component

---

7. In the past three years, have you committed/participated in any of the following types of the study for more than 10 hours per year?

- ☐ Non-management related training
- ☐ Management related training
- ☐ Self-study on management-related topics
- ☐ I did not commit to any of the above training or self-study, but would like to share additional information in relation to my professional development

---

8. Please indicate how important these competencies are to your current management position

	Very unimportant	Somewhat unimportnat	Neither unimportant or important	Somewhat important	Very importa
C1 Evidence-informed decision-making	◯	◯	◯	◯	◯
C2 Operations, administration and resource management	◯	◯	◯	◯	◯
C3 Demonstrated knowledge of veterinary industry environment and the organisation	◯	◯	◯	◯	◯
C4 Interpersonal, communication qualities and relationship management	◯	◯	◯	◯	◯
C5 Leading people and organisation	◯	◯	◯	◯	◯
C6 Enabling and Managing Change	◯	◯	◯	◯	◯

9.

Please indicate whether you felt well prepared to demonstrate each of the following management competencies prior to commencing your current management position.

	Strongly disagree	Disagree	Neither agree nor disagree	Agree	Strongly agree
C1 Evidence-informed decision-making	◯	◯	◯	◯	◯
C2 Operations, administration and resource management	◯	◯	◯	◯	◯
C3 Demonstrated knowledge of veterinary industry environment and the organisation	◯	◯	◯	◯	◯
C4 Interpersonal, communication qualities and relationship management	◯	◯	◯	◯	◯
C5 Leading people and organisation	◯	◯	◯	◯	◯
C6 Enabling and Managing Change	◯	◯	◯	◯	◯

10.

During the past three years, how often have you encountered the following difficulties in fulfilling your management role?

Please add any additional significant difficulties you have encountered not included in the list.

	Never	Rarely	[illegible]
Peer conflict	◯	◯	
Team conflict	◯	◯	
Time constraint	◯	◯	
High staff turnover	◯	◯	
Conflicts with clients	◯	◯	
Creating an innovative team	◯	◯	
Making right hiring decisions	◯	◯	
Losing a high potential employee	◯	◯	
Changing team skill requirements	◯	◯	
Doing something unethical or wrong	◯	◯	
Confronting an employee performance problem	◯	◯	
Inadequate support from senior management of the organisation	◯	◯	
Employee engagement in decision-making and implementation of change	◯	◯	
Having to learn something new such as information or medical technology	◯	◯	
Lack the necessary skills required to fulfill the management responsibilities	◯	◯	
Balancing dual clinical and management responsibilities	◯	◯	

	Never	Rarely	S
Confronting higher management level / dealing with conflicting priorities of senior management	◯	◯	
Others (please provide details)	◯	◯	

11. Please indicate your level of agreement of each of the below 12 items

	Strongly Disagree	Disagree	Neither Agree nor Disagree	Agree	Strongly Agree
The work I do is very important to me	◯	◯	◯	◯	◯
My job activities are personally meaningful to me	◯	◯	◯	◯	◯
The work I do is meaningful to me	◯	◯	◯	◯	◯
I am confident about my ability to do my job	◯	◯	◯	◯	◯
I am self-assured about my capabilities to perform my work activities	◯	◯	◯	◯	◯
I have mastered the skills necessary for my job	◯	◯	◯	◯	◯

12. Please indicate your level of agreement of each of the below 12 items

	Strongly Disagree	Disagree	Neither Agree nor Disagree	Agree	Strongly Agree
I have significant autonomy in determining how I do my job	◯	◯	◯	◯	◯
I can decide on my own how to go about doing my work	◯	◯	◯	◯	◯
I have considerable opportunity for independence and freedom in how I do my job	◯	◯	◯	◯	◯
My impact on what happens in this unit is large	◯	◯	◯	◯	◯

	Strongly Disagree	Disagree	Neither Agree nor Disagree	Agree	Strongly Agree
I have a great deal of control over what happens in this unit	◯	◯	◯	◯	◯
I have significant influence over what happens in this unit	◯	◯	◯	◯	◯

Competency Assessment - 82 behavioral items for five competencies

. Two scales will be used below to measure the level of competence and also the importance of the six competency and 82 associated behavioural items.

**Competency Assessment Descriptive Scale (CADS)**	
CADS 1	May be capable of demonstrating minor aspects in my role
CADS 2	May be capable of demonstrating in my role, but not in all required aspects
CADS 3	Can fully demonstrate in my role with regular guidance
CADS 4	Can generally demonstrate in my role, but guidance is needed occasionally
CADS 5	Can demonstrate in my role independently without guidance
CADS 6	Always apply appropriately in my role with extensive experience
CADS 7	Always apply appropriately in my role, with extensive experience gained from diverse management roles at executive level
Irrelevant	The competency or behavioural item is not so relevant to my management role
**The level of importance to the management role**	
1	Very unimportant
2	Not so important
3	Unsure whether it is important
4	Somewhat important
5	Very important

13. According to the above competency descriptive scale, please indicate the level of competence and importance for each of the following six competencies that you believe you are currently at

	CADS 1	CADS 2	CADS 3	CADS 4	CADS 5	CADS 6
Evidence-informed decision-making	◯	◯	◯	◯	◯	◯
Operations, administration and resource management	◯	◯	◯	◯	◯	◯
Demonstrated knowledge of veterinary industry environment and the organisation	◯	◯	◯	◯	◯	◯
Interpersonal, communication qualities and relationship management	◯	◯	◯	◯	◯	◯
Leading people and organisation	◯	◯	◯	◯	◯	◯

	CADS 1	CADS 2	CADS 3	CADS 4	CADS 5	CADS 6
Enabling and Managing Change	○	○	○	○	○	○

14. According to the above competency descriptive scale, please indicate the level of competence and importance for each of the following six competencies that you believe you are currently at

	Very unimportant	Unimportant	Unsure	Important	i[illegible]
Evidence-informed decision-making	○	○	○	○	
Operations, administration and resource management	○	○	○	○	
Demonstrated knowledge of veterinary industry environment and the organisation	○	○	○	○	
Interpersonal, communication qualities and relationship management	○	○	○	○	
Leading people and organisation	○	○	○	○	
Enabling and Managing Change	○	○	○	○	

14a. According to the above competency descriptive scale, please indicate the level of competence for each of the following behavioral items associating with one of the competencies that you believe you are currently at. If the behavioural item is not relevant to your role, please choose the answer 'N/A'.

	CADS 1	CADS 2	CADS 3	CADS 4	CADS 5	CADS 6
C1.1 Use timely and appropriate questioning/investigation to identify the nature of a problem, issue or opportunity	○	○	○	○	○	○
C1.2 Seek appropriate evidence from multiple organisational sources to guide the identification of solutions	○	○	○	○	○	○
C1.3 Seek appropriate (qualitative /quantitative) evidence from multiple external sources to guide the identification of solutions	○	○	○	○	○	○
C1.4 Critically appraise the validity and relevance of evidence	○	○	○	○	○	○
C1.5 Assess and prioritise the relevance of evidence to the question(s)	○	○	○	○	○	○

14b. According to the above competency descriptive scale, please indicate the level of competence and importance for each of the following behavioral items associating with one of competencies that you

believe you are currently at

	CADS 1	CADS 2	CADS 3	CADS 4	CADS 5	CADS 6
C1.6 Use evidence to question and improve existing practice and processes						
C1.7 Apply the best form(s) of evidence to guide management decision-making						
C1.8 Evaluate the process of seeking and applying evidence to management decision-making						
C1.9 Anticipate decision implementation problems/ impacts and develops and communicate appropriate contingency plans						

14c. According to the above competency descriptive scale, please indicate the level of competence for each of the following behavioral items associating with one of competencies that you believe you are currently at

	CADS 1	2	3	4	5	6	7	Irrelevant
C1.10 Set and use measures to evaluate decision outcomes								
C1.11Support and encourage colleagues and subordinates to use evidence to guide decision-making								
C1.12 Anticipate and prepare for the future by staying abreast of best practice and emerging trends that will have an impact on health outcomes of clients								
C1.13 Commit to ongoing personal and professional development								

14e.

According to the above competency descriptive scale, please indicate the level of competence for each of the following behavioral items associating with one of competencies that you believe you are currently at

	CADS 1	CADS 2	CADS 3	CADS 4	CADS 5	CADS 6
C2.1 Complete necessary workforce records (eg overtime, leave, rosters, attendance, absence) to inform the payroll process						
C2.2 Balance the needs of organisation and of staff through effective planning and management of staff roster and work coverage						
C2.3 Interpret basic financial statements						

	CADS 1	CADS 2	CADS 3	CADS 4	CADS 5	CADS 6
C2.4 Monitor financial performance by analysing a variety of financial data	○	○	○	○	○	○
C2.5 Develop budgets in accordance with organisational objectives	○	○	○	○	○	○
C2.6 Manage budgets in accordance with organisational objectives	○	○	○	○	○	○

14f.

According to the above competency descriptive scale, please indicate the level of competence for each of the following behavioral items associating with one of competencies that you believe you are currently at

	CADS 1	CADS 2	CADS 3	CADS 4	CADS 5	CADS 6
C2.7 Anticipate and plan for changes in policies affecting funding to the organisation/unit	○	○	○	○	○	○
C2.8 Design and develop appropriate roles and reporting structure (across a range of areas) in accordance with organisational objectives	○	○	○	○	○	○
C2.9 Effectively manage recruitment, selection and appointment of sufficient, suitably skilled staff	○	○	○	○	○	○
C2.10 Effectively manage staff turnover and retention	○	○	○	○	○	○
C2.11 Manage staff in accordance with human resource policy and procedure	○	○	○	○	○	○
C2.12 Establish and maintain the organisation's insurance contracts and financial relationships	○	○	○	○	○	○

14g.

According to the above competency descriptive scale, please indicate the level of competence for each of the following behavioral items associating with one of competencies that you believe you are currently at

	CADS 1	CADS 2	CADS 3	CADS 4	CADS 5	CADS 6
C2.13 Conduct regular two-way performance review & development discussions to support staff development	○	○	○	○	○	○
C2.14 Recognise and develop the performance of others by providing timely and appropriate feedback	○	○	○	○	○	○
C2.15 Contribute to continuous improvement of organisational processes, including quality and safety	○	○	○	○	○	○
C2.16 Use performance measures and industry benchmarks to inform continuous performance	○	○	○	○	○	○

	CADS 1	CADS 2	CADS 3	CADS 4	CADS 5	CADS 6
improvement						
C2.17 Plan, execute and evaluate projects with significant scope and impact	○	○	○	○	○	○
C3.1 Demonstrate understanding of the veterinary industry and its impact on veterinary organisations	○	○	○	○	○	○

14h.

According to the above competency descriptive scale, please indicate the level of competence for each of the following behavioral items associating with one of competencies that you believe you are currently at

	CADS 1	CADS 2	CADS 3	CADS 4	CADS 4	CADS 6
C3.2 Demonstrate understanding of political, social, technical and economic factors and their impact on the organisation	○	○	○	○	○	○
C3.3 Demonstrate understanding of the roles of key stakeholders in the veterinary industry and how they interact	○	○	○	○	○	○
C3.4 Demonstrate understanding of the highly professionalised workforce in the veterinary industy	○	○	○	○	○	○
C3.5 Apply relevant legislation and accountability frameworks specific to veterinary settings	○	○	○	○	○	○
C3.6 Demonstrate awareness of clinical and non-clinical risks specific to veterinary organisations	○	○	○	○	○	○
C3.7 Apply quality indices and benchmarks to identify opportunities, set performance standards and improve quality	○	○	○	○	○	○

14i.

According to the above competency descriptive scale, please indicate the level of competence for each of the following behavioral items associating with one of competencies that you believe you are currently at

	CADS 1	CADS 2	CADS 3	CADS 4	CADS 5	CADS 6
C3.8 Apply risk management concepts and techniques in their work	○	○	○	○	○	○
C3.9 Demonstrate understanding of the diversity of veterinary needs	○	○	○	○	○	○
C3.10 Demonstrate awareness of the organisation's history, culture and development	○	○	○	○	○	○

	CADS 1	CADS 2	CADS 3	CADS 4	CADS 5	CADS 6
C3.11 Effectively navigate organisational structures, roles and relationships in order to achieve work goals						
C4.1 Show trust and respect for others in their actions						
C4.2 Provide appropriate support to others in the workplace						
C4.3 Listen and empathise with others						
C4.4 Engage confidently and constructively in verbal and non-verbal interactions with others						

14j.

According to the above competency descriptive scale, please indicate the level of competence for each of the following behavioral items associating with one of competencies that you believe you are currently at

	CADS 1	CADS 2	CADS 3	CADS 4	CADS 5	CADS 6	[illegible]
C4.5 Communicate verbally in a clear, logical and grammatically correct manner in both formal and informal situations							
C4.6 Produce written reports/materials which are appropriate for both audience and purpose							
C4.7 Invest time and effort in working and engaging with stakeholders							
C4.8 Actively question, listen, respond and provide feedback as a basis for effective communication							
C4.9 Function effectively in a team by developing and maintaining professional relationships with people from a wide range of backgrounds							
C4.10 Build collaborative internal and external relationships							

14k.

According to the above competency descriptive scale, please indicate the level of competence for each of the following behavioral items associating with one of competencies that you believe you are currently at

	CADS 1	CADS 2	CADS 3	CADS 4	CADS 5	CADS 6	[illegible]
C4.11 Adopt a flexible, client-oriented approach that is sensitive to diverse needs							
C4.12 Consider and act with sensitivity to the politics of any given situation							

	CADS 1	CADS 2	CADS 3	CADS 4	CADS 5	CADS 6	(
C4.13 Work through conflict (and with diverse views) by initiating and engaging in robust conversations							
C4.14 Demonstrate awareness of own emotions and their impact on others							
C4.15 Show self-control over disruptive emotions and impulses							
C4.16 Maintain focus, set realistic goals and are not easily distracted							

14l.

According to the above competency descriptive scale, please indicate the level of competence for each of the following behavioral items associating with one of competencies that you believe you are currently at

	CADS 1	CADS 2	CADS 3	CADS 4	CADS 5	CADS 6	(
C4.17 Show awareness of, and sensitivity to, the feelings of others							
C4.18 Invest time in self-care and personal support mechanisms, especially during stressful times.							
C4.19 Promote and adhere to high standards for personal and organisational integrity, honesty, transparency and respect for people							
C5.1 Develop and/or implement a shared vision to achieve organisational goals							
C5.2 Manage with reference to the broader organisational context							
C5.3 Engage effectively in organisational decision-making							

14m.

According to the above competency descriptive scale, please indicate the level of competence for each of the following behavioral items associating with one of competencies that you believe you are currently at

	CADS 1	CADS 2	CADS 3	CADS 4	CADS 5	CADS 6	(
C5.4 Inform and educate influential decision makers							
C5.5 Balance the values and priorities of both organisation and profession(s)							
C5.6 Lead, develop and evaluate performance to build an effective team							

	CADS 1	CADS 2	CADS 3	CADS 4	CADS 5	CADS 6	(
C5.7 Empower others to achieve goals	○	○	○	○	○	○	
C5.8 Adapt leadership style to suit the situation	○	○	○	○	○	○	
C5.9 Establish and maintains a personal and professional support network	○	○	○	○	○	○	

14n.

According to the above competency descriptive scale, please indicate the level of competence for each of the following behavioral items associating with one of competencies that you believe you are currently at

	CADS 1	CADS 2	CADS 3	CADS 4	CADS 5	CADS 6	(
C5.10 Persevere to achieve goals	○	○	○	○	○	○	
C5.11 Demonstrate energy, commitment and enthusiasm	○	○	○	○	○	○	
C5.12 Encourage ideas and identify opportunities	○	○	○	○	○	○	
C5.13 Remain calm whilst under pressure	○	○	○	○	○	○	
C6.1 Explain the need for change in an effective way	○	○	○	○	○	○	
C6.2 Assess readiness for change and plans accordingly	○	○	○	○	○	○	

14o.

According to the above competency descriptive scale, please indicate the level of competence for each of the following behavioral items associating with one of competencies that you believe you are currently at

	CADS 1	CADS 2	CADS 3	CADS 4	CADS 5	CADS 6	C
C6.3 Act accountably and accept personal responsibility	○	○	○	○	○	○	
C6.4 Effectively balance consultation and decisiveness in decision-making	○	○	○	○	○	○	
C6.5 Use available evidence to appraise options	○	○	○	○	○	○	
C6.6 Anticipate and appreciate the impact of change and plans accordingly	○	○	○	○	○	○	
C6.7 Implement change and effectively manage the transition process	○	○	○	○	○	○	
C6.8 Evaluate the processes and outcomes of change	○	○	○	○	○	○	
C6.9 Recognise and tolerate ambiguity	○	○	○	○	○	○	

15. What are the career goals that you would like to achieve within the next five years? How can the organization best support you in achieving your career goals?

16. Please share the thoughts about the best ways of improving your managerial competence

17. Have you identified obstacles in demonstrating your managerial competence in your current management role? What are they?

18. What support would you like to receive from your organisation to help you to perform your job better and more easily?

Submission .

You have come to the end of the assessment. Please click Yes to submit the answers, or use the previous button to review the answers.

○ Yes

Powered by Qualtrics
